# COVID-19 Misinformation Spread in Eight Countries: Exponential Growth Modeling Study

**DOI:** 10.2196/24425

**Published:** 2020-12-15

**Authors:** Elaine Okanyene Nsoesie, Nina Cesare, Martin Müller, Al Ozonoff

**Affiliations:** 1 Department of Global Health School of Public Health Boston University Boston, MA United States; 2 Biostatistics and Epidemiology Data Analytics Center School of Public Health Boston University Boston, MA United States; 3 Digital Epidemiology Lab, École Polytechnique Fédérale Geneva Switzerland; 4 Department of Pediatrics Harvard Medical School Boston, MA United States

**Keywords:** misinformation, internet, COVID-19, social media, rumors

## Abstract

**Background:**

The epidemic of misinformation about COVID-19 transmission, prevention, and treatment has been going on since the start of the pandemic. However, data on the exposure and impact of misinformation is not readily available.

**Objective:**

We aim to characterize and compare the start, peak, and doubling time of COVID-19 misinformation topics across 8 countries using an exponential growth model usually employed to study infectious disease epidemics.

**Methods:**

COVID-19 misinformation topics were selected from the World Health Organization Mythbusters website. Data representing exposure was obtained from the Google Trends application programming interface for 8 English-speaking countries. Exponential growth models were used in modeling trends for each country.

**Results:**

Searches for “coronavirus AND 5G” started at different times but peaked in the same week for 6 countries. Searches for 5G also had the shortest doubling time across all misinformation topics, with the shortest time in Nigeria and South Africa (approximately 4-5 days). Searches for “coronavirus AND ginger” started at the same time (the week of January 19, 2020) for several countries, but peaks were incongruent, and searches did not always grow exponentially after the initial week. Searches for “coronavirus AND sun” had different start times across countries but peaked at the same time for multiple countries.

**Conclusions:**

Patterns in the start, peak, and doubling time for “coronavirus AND 5G” were different from the other misinformation topics and were mostly consistent across countries assessed, which might be attributable to a lack of public understanding of 5G technology. Understanding the spread of misinformation, similarities and differences across different contexts can help in the development of appropriate interventions for limiting its impact similar to how we address infectious disease epidemics. Furthermore, the rapid proliferation of misinformation that discourages adherence to public health interventions could be predictive of future increases in disease cases.

## Introduction

SARS-CoV-2 has infected more than 18.4 million people worldwide and has resulted in approximately 692,000 deaths [1]. Fast-paced research intended to understand the disease biology and dynamics, the novelty of the pandemic experience, and the quickly evolving physical distancing protocols have meant rapid changes in the public’s understanding of the disease, which has created an environment primed for the spread of misinformation. These include unsubstantiated or false claims that typically relate to one of four topics: transmission, prevention, vaccination, and treatment [2]. For example, there have been claims that COVID-19 was originally developed as a bioweapon [3] and false information about preventive substances or remedies including vitamin C and D, zinc, elderberry, chlorine dioxide, silver, and essential oils [4,5]. The need for information; anxiety about physical, social, and economic impacts of the virus; and a lack of a centralized authority available to detect and combat misinformation created an environment in which false assertions about COVID-19 could spread unchecked [6,7]. Although institutional efforts have been made to combat false claims about COVID-19 through channels such as the World Health Organization (WHO) Mythbusters website [8] and the Food and Drug Administration’s Health Fraud Press Announcements [5], the control of misinformation remains a challenge [2].

Although tracking the origins and spread of false beliefs surrounding COVID-19 remains difficult, infodemiology [9,10] may provide a framework for tracking and analyzing social determinants of COVID-19 misinformation spread [11-13]. Moreover, there is evidence that trends in how misinformation spreads online parallels the spread of epidemics [14,15]. We therefore aim to understand how misinformation exposure differs across countries, what similarities and differences exist, and what types of misinformation spread fastest. We used epidemic modeling techniques to characterize misinformation about COVID-19 in 8 countries, focusing on the start, peak, and the doubling time of searches. Characterizing how misinformation- seeking trends develop online can be useful in the design of appropriate interventions that aid in the control of epidemics.

## Methods

### Data

We constructed a term list consisting of a combination of “Coronavirus,” “COVID-19,” “COVID19,” and “COVID” with misinformation terms obtained from the WHO Mythbusters website [8]: wine, hot weather, antibiotics, chlorine, garlic, ginger, sun, 5G, hydroxychloroquine, pepper, houseflies, mosquito, hand dryer, supplement, and saline. We selected topics that were clearly defined and for which there was available data. For example, someone searching for “COVID AND Alcohol” might be interested in the amount of alcohol needed to make hand sanitizers at home and not necessarily trying to verify the claim that drinking alcohol might cure or prevent COVID-19. Topics such as 5G are reported to have spread quickly through social networks on Twitter [16]. Additionally, searches for terms such as mosquito and hand dryers were prevalent in some countries and completely absent in others.

After assessing the quality of search data across the countries, we focused on four misinformation topics: claims that (1) drinking alcohol (specifically, wine) increases immunity to COVID-19; (2) sun exposure prevents spread or that COVID-19 is less likely to spread in hot, sunny areas; (3) home remedies may prevent or cure COVID-19; and (4) COVID-19 is spread via 5G cellular networks. We also discussed searches for hydroxychloroquine separately because, unlike the other misinformation terms, it did not appear on the WHO website until July 31, 2020. There was also much confusion about its potential benefit while it was being evaluated by clinicians, unlike the other misinformation topics.

We focused on 8 English-speaking countries from five continents: Nigeria, Kenya, South Africa, the United States, the United Kingdom, India, Australia, and Canada. Weekly search data was obtained from the Google Trends application programming interface from December 2019 to October 2020.

### Analysis

We assumed the search data represented trends in exposure to misinformation. This implies that if someone is seeking information on a particular misinformation topic, they have been exposed to it. However, we cannot deduce a person’s intent or whether or not they believe the misinformation. Additional data is needed to deduce the personal motives of individuals engaging in searches (see Discussion section). We inferred the week of the first peak and then fitted an exponential growth model to both sides of the time series curve: before and after the peak. The exponential regression model is defined as follows: log(*y*) = *r* × *t* × *b*, where *y* represents searches (or postings) for the misinformed phrase, and *r*, *t*, and *b* represent the growth rate, number of days since Google reported a search volume greater than one, and the intercept, respectively. This approach was implemented in the Incidence package in the R software (R Foundation for Statistical Computing) and used for analyzing incidence data for epidemics [17,18]. We compared the start week, doubling time, and first peak across the 8 countries and four topics. We referred to the peak of the search data as the initial peak, since similar to an epidemic, there can be multiple peaks. New searches might be initiated at a later time during the pandemic.

## Results

### Start and Peak Weeks

**Figure 1:**
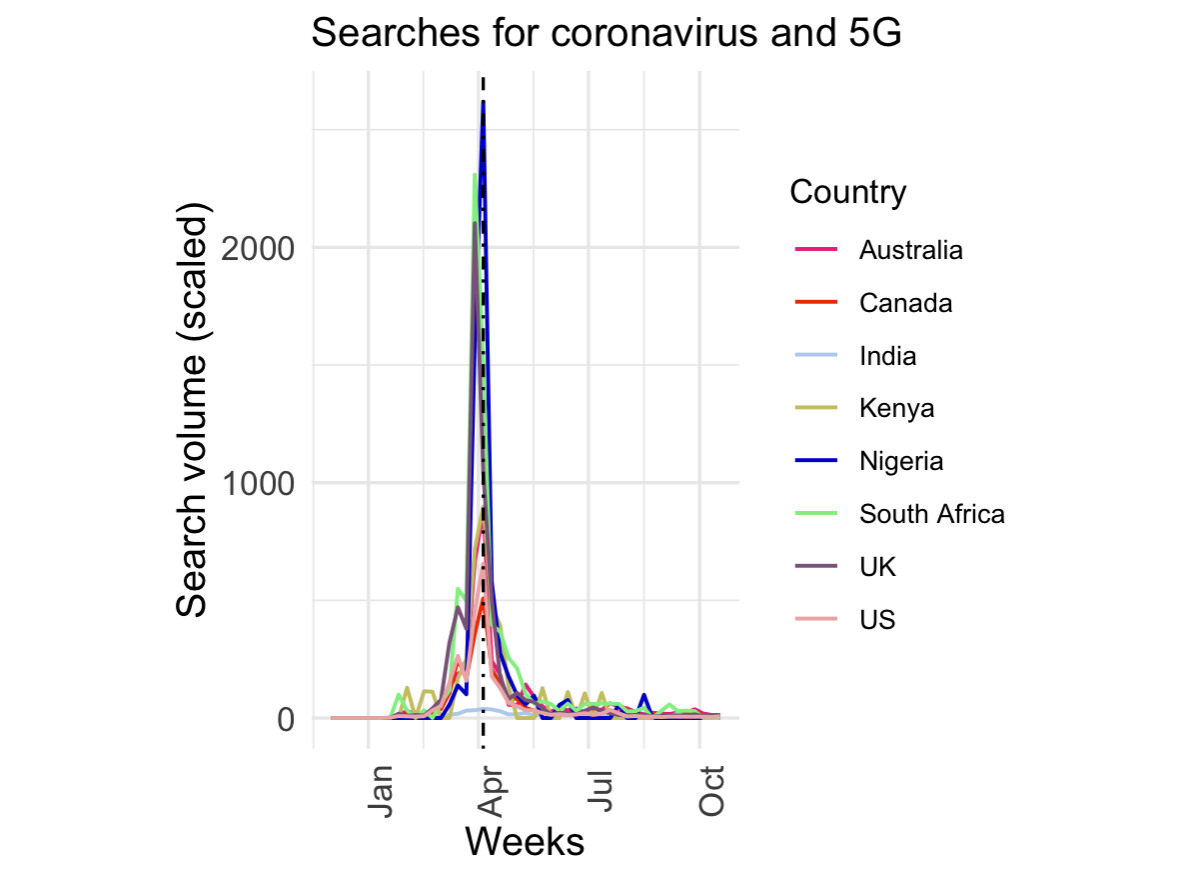
Trends in searches for coronavirus and 5G across 8 countries from December 2019 to October 2020. The black vertical line indicates the time the World Health Organization included the topic on the Mythbusters website.

Searches for “coronavirus AND 5G” started at different times but peaked in the same week for 6 of the countries (Figure 1). For example, searches for 5G in Australia, the United Kingdom, and Canada were first reported during the week of January 19, 2020, while searches in South Africa, India, and the United States started the following week. In contrast, searches were recorded from Kenya and Nigeria a month later, during the weeks of February 16 and 23, respectively. Despite the different start dates, India, Australia, Canada, Kenya, Nigeria, and the United States observed the first search peak during the week of April 5, 2020. The United Kingdom and South Africa observed a peak during the same week: March 29, 2020.

Similarly, searches for “coronavirus AND ginger” started in the same week in several countries. The United States, the United Kingdom, Canada, Australia, and India noted initial searches during the week of January 19, 2020. However, initial searches in South Africa, Nigeria, and Kenya occurred several weeks later, during the weeks of February 9, February 23, and March 8, respectively. The peak week was earliest for the United Kingdom (March 22), followed by Canada (March 29) and the United States and Australia (April 5). South Africa, Nigeria, India, and Kenya peaked during the weeks of April 12, April 12, April 19, and April 26, respectively. Searches did not always grow exponentially after the initial week. For some countries such as Nigeria, zeros were noted during 1 or 2 consecutive weeks after the initial search. This might be due to Google’s scaling algorithm and might not represent no searches during those weeks.

Furthermore, searches for “coronavirus AND sun” started in the United States, Canada, Australia, India, and the United Kingdom during the week of January 19, 2020, in Nigeria and South Africa during the week of January 26, and a month later in Kenya (February 23). Multiple countries noted a peak during the same week: the week of March 15 for the United States, South Africa, and Canada; March 22 for Australia, the United Kingdom, and Nigeria; and April 12 for India and Kenya.

Lastly, search trends for “coronavirus AND wine” were inconsistent across the 8 countries. Nigeria and Kenya had low search volume and were therefore excluded. The United States noted the earliest searches during the week of January 12, 2020. Other countries noted initial search during the weeks of January 19 (Canada, India, the United Kingdom), January 26 (Australia), and February 9 (South Africa). The peak weeks also differed across the countries with no obvious groupings across regions. The peak weeks were March 15 (Canada and South Africa), March 22 (the United Kingdom), April 5 (Australia and the United States), and April 12 (India).

### Doubling Time

**Figure 2:**
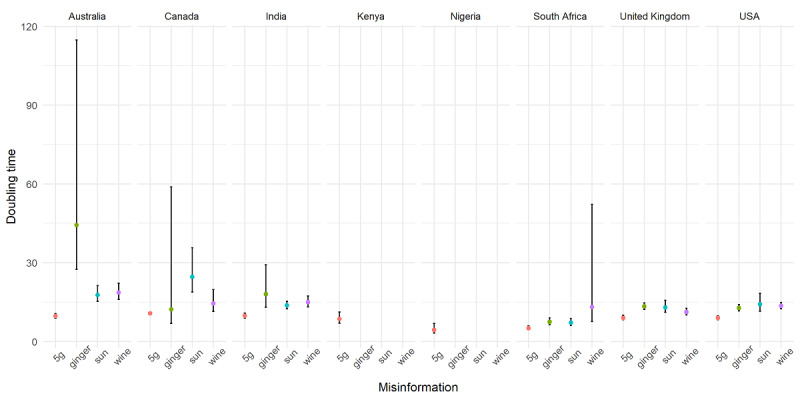
The estimated doubling time for searches on four misinformation topics: 5G, wine, ginger, and sun.

Searches for 5G had the shortest doubling time across all misinformation topics (Figure 2). Nigeria and South Africa noted the shortest doubling time: between 4 and 5 days. Searches for ginger doubled at approximately the same rate for the United Kingdom and the United States. Searches for sun doubled much more slowly in Canada compared to the other countries. The confidence interval was also wider, suggesting sparse searches in these contexts and less confidence in the estimates. Similar observations were noted for searches of ginger in Australia. Searches for wine were more prevalent in the United Kingdom, the United States, India, and Australia. The data for Kenya, Nigeria, Canada, and South Africa were sparse.

## Discussion

Despite access to the same social media and search platforms, exposure to misinformation appeared different across the 8 countries included in our study. Searches for the majority of misinformation topics varied in start and peak time, and did not necessarily grow exponentially.

These differences in the timing of initial searches could be due to disparities in access, culture, and how internet platforms are used in different parts of the world. Moreover, the sparseness of searches for topics such as ginger in some contexts indicates contextual differences in the concerns and interests.

**Figure 3:**
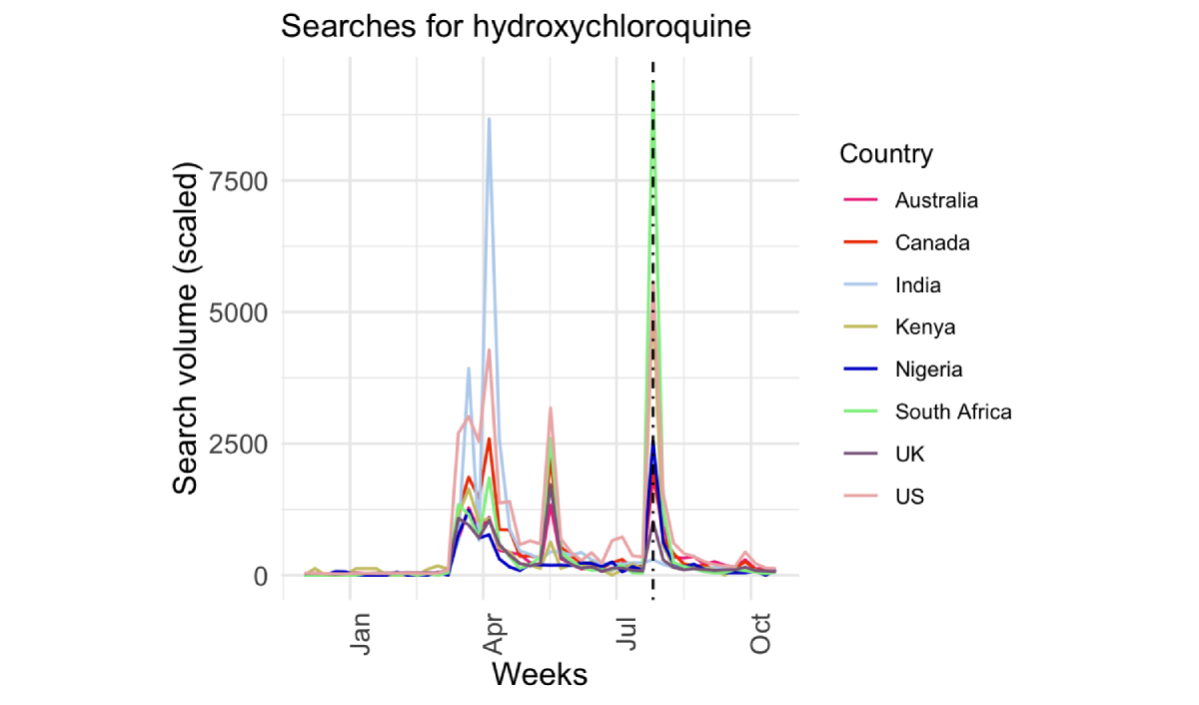
Trends in searches for hydroxychloroquine across 8 countries from December 2019 to October 2020. The black vertical line indicates the time the World Health Organization included the topic on the Mythbusters website.

Searches for 5G and hydroxychloroquine displayed unique patterns that cut across contexts. Not only did searches for 5G have the fastest doubling time, but they also started and peaked around the same time for most of the countries. Searches for hydroxychloroquine had a distinct trend when compared to the other topics (Figure 3), owing to the public and medical discussion of its potential benefits over several months. The WHO listed hydroxychloroquine on their website on July 31, 2020, during what appears to be the second or third wave of searches.

Misinformation about COVID-19 transmission, prevention, and treatment can impact how the public reacts to public health interventions such as wearing a mask and social distancing, which can lead to an uptick in reported cases. Although it is beyond the scope of this paper to link specific misinformation topics to the spread of COVID-19 in different countries, we observed that, for some misinformation topics such as 5G, the official date of the WHO’s response noted on the Mythbusters website appeared after the first peak in searches for some countries (Figure 1). This was similar for the other misinformation topics. In addition, we did not observe a resurgence in 5G searches after the first peak. This observation supports the idea that timely response from trusted public health sources are needed to counter the spread of misinformation and that digital platforms may be useful tools that the WHO and other organizations may combat such falsehoods.

Research that combines data from multiple digital platforms is needed to comprehensively study the emergence of various misinformation topics and their association with reports of increased COVID-19 activity in various regions. These studies must be mindful of context, however, given differences in testing capacity, case, and mortality reporting across countries. Nonetheless, such studies could improve our understanding of the potential impact of misinformation on the transmission of SARS-CoV-2. Additionally, future studies may gather behavioral data to assess how the rapid proliferation of misinformation discourages adherence to public health interventions related to COVID-19. These studies must also take into account the culture, policies, and regional variation of each country analyzed.

We acknowledge limitations in our data. First, only people who have access and can afford using the internet are likely to spend time investigating these misinformation topics. This therefore leaves out a large percentage of the population. The percentage of the population within each country that used the internet in 2017 varied significantly among study contexts, ranging from more than 90% in the United Kingdom to less than 10% in Nigeria [19]. Second, the keyword selection and phrases that characterized our data collection may have unintentionally omitted relevant content or included noise. Third, an analysis of the network characteristics of individuals involved in spreading misinformation would better inform intervention strategies.

Identifying where misinformation trends emerge and how quickly they spread can be used to direct crisis communication and provide more effective health care [10]. This study illustrates that neighboring countries can have different misinformation experiences related to similar topics, which can impact control of COVID-19 in these countries. Although monitoring misinformation-seeking behavior via Google Trends is one pathway for identifying belief prevalence and trends, we should monitor information flow across multiple platforms including social media sites such as Facebook, Twitter, and Instagram, and messaging apps such as WhatsApp.

## Abbreviations

WHO: World Health Organization
